# Relationship between kinetic parameters of ultrafast dynamic contrast-enhanced (DCE) MRI and tumor-infiltrating lymphocytes (TILs) in breast cancer

**DOI:** 10.1007/s11604-024-01645-w

**Published:** 2024-08-26

**Authors:** Ken Yamaguchi, Takahiko Nakazono, Ryoko Egashira, Shuichi Fukui, Tsutomu Imaizumi, Katsuya Maruyama, Dominik Nickel, Takahiro Hamamoto, Rin Yamaguchi, Hiroyuki Irie

**Affiliations:** 1 Department of Radiology, Faculty of Medicine, Saga University, 5-1-1 Nabeshima, Saga, 849-8501 Japan; 2 MR Research & Collaboration Department, Siemens Healthcare K.K., Gate City Osaki West Tower, 1-11-1 Osaki, Shinagawa-Ku, Tokyo, 141-8644 Japan; 3 MR Application Development, Siemens Healthcare GmbH, Allee Am Roethelheimpark 2, 91052 Erlangen, Germany; 4 Ajisai Clinic, 1-9-38 Ekimaechuo, Saga, 840-0801 Japan; 5 Department of Pathology and Laboratory Medicine, Kurume University Medical Center, 155-1 Kokubu, Kurume, 859-0863 Japan

**Keywords:** Breast cancer, Magnetic resonance imaging, Ultrafast, TILs, Prognosis

## Abstract

**Purpose:**

To evaluate the relationship between kinetic parameters of ultrafast dynamic contrast-enhanced (DCE) magnetic resonance imaging (MRI) and tumor-infiltrating lymphocytes (TILs) in breast cancer.

**Patients and methods:**

This retrospective study was approved by an institutional review board and included 76 women (median age: 60) with 76 surgically proven breast cancers who underwent DCE MRI including ultrafast sequence. Based on the TILs level, we classified the patients into the low-TILs (< 10%) group and the high-TILs (≥ 10%) group. Maximum slope (MS) and time to enhancement (TTE) derived from ultrafast DCE sequence were correlated in each TILs group. The percentages of six kinetic patterns (fast, medium, and slow from the early phase, washout, plateau, and persistent from the delayed phase) derived from the conventional DCE sequence were also correlated in each TILs group.

**Results:**

Of the 76 breast cancers, 57 were in the low-TILs group and 19 comprised the high-TILs group. The median MS in the high-TILs group (32.4%/sec) was significantly higher than that in the low-TILs group (23.68%/s) (*p* = 0.037). In a receiver-operating characteristic (ROC) analysis, the area under the curve (AUC) for differentiating between the high- and low-TILs group was 0.661. The TTE in the high-TILs group was significantly shorter than that in the low-TILs group (*p* = 0.012). In the ROC analysis, the AUC was 0.685. There were no significant differences between the percentages of the six kinetic patterns from the conventional DCE sequence and the TILs level (*p* = 0.075–0.876).

**Conclusion:**

Compared to the low-TILs group, the high-TILs group had higher MS and shorter TTE.

## Introduction

Breast cancer is the most frequent cancer in women worldwide [1]. Some breast cancers are rich in lymphocytes and plasma cells, and these cancers are known to have a good prognosis [2]. Tumor-infiltrating lymphocytes (TILs) are a new biomarker for the prognosis and the chemotherapy response in breast cancer. Several research groups have reported that patients with breast cancer with abundant TILs showed good survival outcomes and good chemotherapy responses, especially the patients with triple-negative breast cancer (TNBC) and human epidermal growth factor receptor-2 (HER2)-positive breast cancer [3–5]. Previously, the clinical significance of TILs in estrogen receptor (ER)-positive breast cancer has not been established [5]. However, recently, there is a study reporting good distant disease-free survival in TILs-rich ER-positive breast cancer patients with high ki67 marker who underwent neoadjuvant chemotherapy [6]. The value of TILs in ER-positive breast cancer is recently being investigated.

At the preoperative stage of breast cancer, an evaluation of TILs should be done using a biopsy specimen, but this may not reflect the whole breast cancer TILs status. In contrast, breast magnetic resonance imaging (MRI) can evaluate whole breast cancer characteristics. There are many studies for predicting TILs level [7–16]. In these studies, morphological characteristics [7, 10], kinetic parameters of the standard dynamic contrast-enhanced (DCE) sequence [8, 10, 13], the apparent diffusion coefficient (ADC) value of diffusion-weighted imaging (DWI) [9–11], peritumoral edema in T2-weighted imaging (WI) [12], and radiomics parameters [14–16] were correlated with the level of TILs. In these studies, high levels of TILs are associated with higher ADC value [9, 10], round shape and circumscribed margin [7], peritumoral edema in T2WI [12], and radiomics parameters [14–16]. High levels of TILs are also associated with lower percentages of the persistent pattern [8] and lower peak enhancement [13] in standard DCE sequence. Radiomics analysis is complicated and standard DCE sequence takes 4 to 5 min to get the washout pattern which suggests typical malignant pattern. It would be useful to have a method that could predict the TILs status through easy analysis and short time image acquisition.

Ultrafast DCE MRI is a dynamic study that can acquire images with very short scan times at a very early post-contrast phase [17]. Ultrafast DCE MRI was initially designed to differentiate between benign and malignant disease [18–20], but in recent years, its associations with pathologic or prognostic factors in breast cancer have also been investigated [21–24]. However, to our knowledge, there is no published study that directly investigated TILs level with the findings of ultrafast DCE MRI. Although standard DCE MRI is currently very useful for predicting TILs status, if ultrafast DCE MRI can predict the TILs level in breast cancer, it would be useful because of its short scan time compared to standard DCE MRI in the future. We conducted the present study to evaluate the relationship between kinetic parameters of ultrafast DCE MRI and TILs in breast cancer.

## Materials and methods

This retrospective study was approved by the institutional review board, and the requirement of written informed consent was waived.

### Patients

**Fig. 1:**
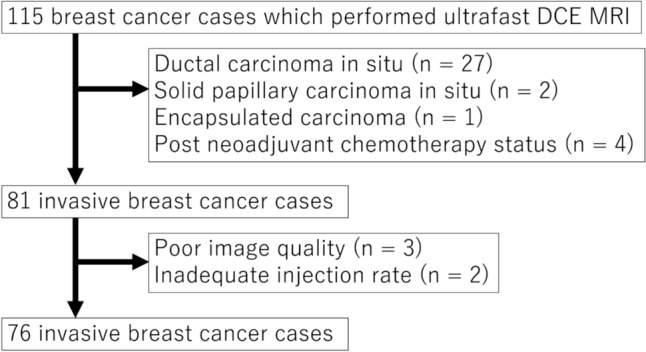
Flowchart of case selection

From August 2018 to July 2021, a total of 115 breast cancer patients underwent breast DCE MRI including ultrafast DCE using a prototypical volume-interpolated breath-hold examination (VIBE) sequence with compressed sensing (CS) reconstruction under free breathing at our institution after a request for preoperative breast MRI from a breast care practitioner. These 115 cases were candidates for this study, because the TILs status in each case was easily evaluated by an experienced breast pathologist at a later date. Of these, we excluded the cases with in situ cancer (ductal carcinoma in situ, solid papillary carcinoma in situ, and encapsulated papillary carcinoma), cases after neoadjuvant chemotherapy, and cases with an inadequate injection rate. In addition, lesions with significant disturbance in the time intensity curve were excluded as cases of poor image quality, because they were considered to be largely influenced by movement. The flowchart of case selection is shown in Fig. 1.

### MRI technique

All examinations were performed using a 3 T MR system (MAGNETOM Prisma, Siemens Healthcare, Erlangen, Germany) with a dedicated 18-channel breast coil. For the ultrafast DCE imaging, one phase was acquired before the contrast injection and 30 phases after the contrast injection (the 2nd phase was started together with the contrast injection) with 2.9-s temporal resolution (the total scan time after the contrast material injection for the ultrafast DCE sequence was 87 s) using a prototypical VIBE sequence with CS reconstruction under free breathing. The contrast material (0.1 mmol/kg of gadobutrol; Gadovist, Bayer Schering Pharma, Berlin, Germany) was injected intravenously, followed by a 20 mL saline flush at a rate of 2.5 mL/sec.

Before the ultrafast DCE sequence, axial diffusion-weighted images based on readout-segmented echoplanar imaging (EPI), axial spin echo T1-weighted images, fat-suppressed axial spin echo T2-weighted images, and one pre-contrast-enhanced fat-suppressed axial T1-weighted image using a VIBE sequence for a conventional DCE study were obtained. After the ultrafast DCE sequence, two contrast-enhanced fat-suppressed axial T1-weighted images using VIBE sequences for the standard DCE study and one contrast-enhanced fat-suppressed sagittal T1-weighted images using VIBE sequences as high spatial resolution images were obtained. First post-contrast axial T1-weighted images were started to scan just after finished ultrafast DCE sequence and second post-contrast axial T1-weighted images were started to scan after 270 s of contrast material injection. High-resolution sagittal T1-weighted images were scanned between first and second post-contrast DCE sequences. Our breast MRI parameters are provided in Table 1.

**Table 1:** MRI parameters

	DWI	T2WI with FS	T1WI	Ultrafast DCE study with FS (31 series)	Conventional DCE study with FS (1 pre and 2 series)	High-resolution image
Orientation	Axial	Axial	Axial	Axial	Axial	Sagittal
Sequence	rs-EPI	TSE	TSE	CS	VIBE	VIBE
TR/TE, ms	8190/41	4900/74	500/9.4	3/1.2	4/1.5	3.73/1.45
Flip angle	90	120	160	12	10	10
Echotrain length	0	9	3	1	1	1
Matrix	160 × 56	448 × 269	512 × 307	384 × 269	384 × 326	384 × 288
Thickness, mm	3.0	3.0	3.0	2.5	0.9	0.7
Voxel size, mm	2.1 × 2.1 × 3.0	0.8 × 0.8 × 3.0	0.7 × 0.7 × 3.0	0.9 × 0.9 × 2.5	0.8 × 0.8 × 0.9	0.7 × 0.7 × 0.7
Acquisition time, sec	229	201	88	2.9/series	60/series	120

*DWI* diffusion-weighted image, *T2WI* T2-weighted image, *FS* fat suppression, *T1WI* T1-weighted image, *DCE* dynamic contrast-enhanced, *rs-EPI* readout-segmented echoplanar image, *TSE* turbo spin echo, *CS* compressed sensing, *VIBE* volume-interpolated breath-hold examination

### Postprocessing

The ultrafast images were created on the MRI console. Each lesion was automatically segmented and color-coded. In these lesions, the region of interest (ROI) of highest enhancement at the last phase of the ultrafast DCE image was automatically identified, and the time intensity curve of this ROI was automatically calculated. The size of each ROI was fixed at 5 pixels (4.91 mm^2^). For analysis, maximum slope (MS) and time to enhancement (TTE) were used. The MS (%/sec) was defined as the slope of the tangent at the steepest part of the time intensity curve of the ultrafast DCE MRI, as described [18], and MS was calculated based on the time intensity curve. The TTE was defined as the time point at which the lesion starts to enhance minus the time point at which the descending aorta starts to enhance [25]. TTE was calculated using multiwindow of the workstation. We also calculated the percentages of six kinetic patterns derived from the conventional DCE sequence: (i) fast, (ii) medium, and (iii) slow from the early phase and (iv) washout, (v) plateau, and (vi) persistent from the delayed phase. The percentages of these six kinetic patterns were calculated from the entire tumor volume automatically extracted by the workstation. These MS values, TTE values derived from the ultrafast DCE sequence, and the percentages of the six kinetic patterns derived from the conventional DCE sequence are routinely calculated in daily clinical practice. A dedicated workstation (PMview; JMAC, Sapporo, Japan) was used for these calculations.

### Analyses

Each patient's TILs status was evaluated by a single experienced breast pathologist (R.Y.) according to the recommendations of the International Immuno-Oncology Biomarker Working Group on Breast Cancer [26]. TILs were scored in 10% increments (< 10%, 10%, 20%, 30%…). Correlations of distribution of TILs between MS and TTE were analyzed. The cut-off value of TILs score has been set in various ways and has not been clearly established [7–16]. Therefore, as has been adopted in some studies, we set the cut-off value at 10% to separate the low group and, because the number of cases was small, those above 10% were set as the high group [9, 10, 14, 15]. After the pathological evaluation, we classified the patients into the low-TILs group (< 10%) or the high-TILs group (≥ 10%). Other pathological findings were collected from the patients' postoperative pathological reports and correlated each TILs group.

MS and TTE were correlated with each TILs group. The percentages of the six kinetic patterns derived from the standard DCE sequence were also correlated with each TILs group. Due to the small number of cases and datasets, the results were not normally distributed according to Shapiro–Wilk test. The Wilcoxon rank-sum test, the χ^2^-test, and Fisher's exact test were used for statistical evaluation. Probability (p) values < 0.05 were considered significant. When we observed a significant difference in kinetic parameters, we performed a receiver-operating characteristic (ROC) analysis. JMP software (ver. 16.1.0, SAS, Tokyo) was used for all statistical analyses.

## Results

The cases of a total of 76 patients (median age: 60 years) with 76 invasive breast cancers were collected for this study. Of these, 57 cases were scored as < 10%, 6 cases scored as 10%, 8 cases scored as 20%, 3 cases scored as 30%, and 2 cases scored as 40%. TILs were significantly positive correlated with MS (*p* = 0.028) and negative correlated with TTE (*p* = 0.039). Of these, 57 cases were evaluated as breast cancer with a low level of TILs, and the other 19 cases were evaluated as breast cancer with a high level of TILs. The results of our analyses of the potential relationships between the various pathological findings and their TILs status are summarized in Table 2. There were significant correlations between the TILs status and the following: the hormone receptor status, HER2 status, a proliferative marker (the Ki67 index), and histological grade. The high-TILs group tended to have a negative hormone receptor status, a negative HER2 status, higher Ki67 index values, and high histological grades.

**Table 2:** Relationships between various pathological findings and TILs status

		Low TILs *n* = 57	High TILs *n* = 19	*P* value
Age*		61 (36–79)	58 (28–78)	0.2566
Menopausal status	pre	16	9	0.121
	post	41	10	
Size, mm*		21 (6–100)	19 (4–60)	0.951
ER	Positive	55	12	0.0006
	Negative	2	7	
PgR	Positive	48	9	0.0042
	Negative	9	10	
HER2	Positive	2	5	0.0092
	Negative	55	14	
Ki67*		10 (1–30) %	20 (2–85) %	0.0068
Histological grade	1	20	6	0.0002
	2	35	5	
	3	2	8	
LN metastasis	Positive	8	3	1.0000
	Negative	49	16	

^*^Values are median (range). *ER* estrogen receptor, *PgR* progesterone receptor, *HER2* human epidermal growth factor receptor-2, *LN* lymph node, *TIL* tumor-infiltrating lymphocytes

**Fig. 2:**
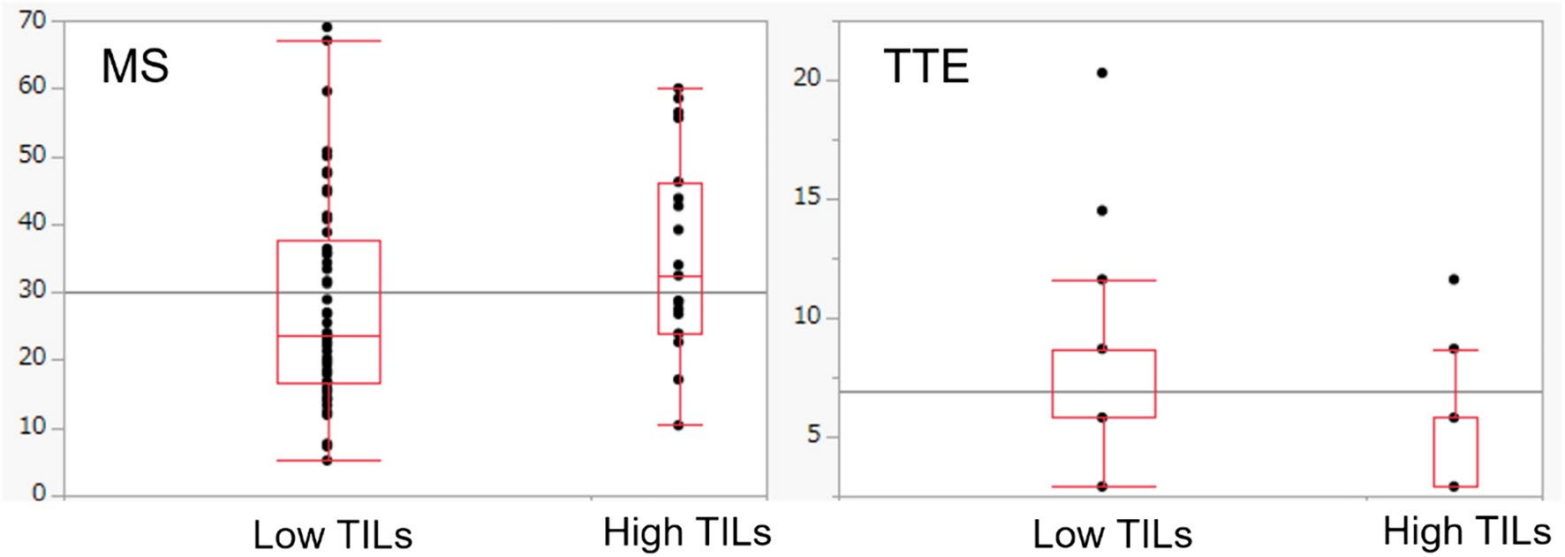
Boxplots of the maximum slope (MS) and time to enhancement (TTE). Compared to the low-TILs group, the high-TILs group had significantly higher MS and shorter TTE. TILs: tumor-infiltrating lymphocytes

The relationships between TILs status and their MS and TTE results are presented in Table 3 and Fig. 2.

**Table 3:** The relationships between MS, TTE, and TILs status

	Low TILs *n* = 57	High TILs *n* = 19	*P* value
MS	23.68 (5.19–69.05)	32.4 (10.36–60)	0.0369
TTE	5.8 (2.9–20.3)	5.8 (2.9–11.6)	0.0122

Values are presented as median (range). *MS* maximum slope, *TILs* tumor-infiltrating lymphocytes, *TTE* time to enhancement

**Fig. 3:**
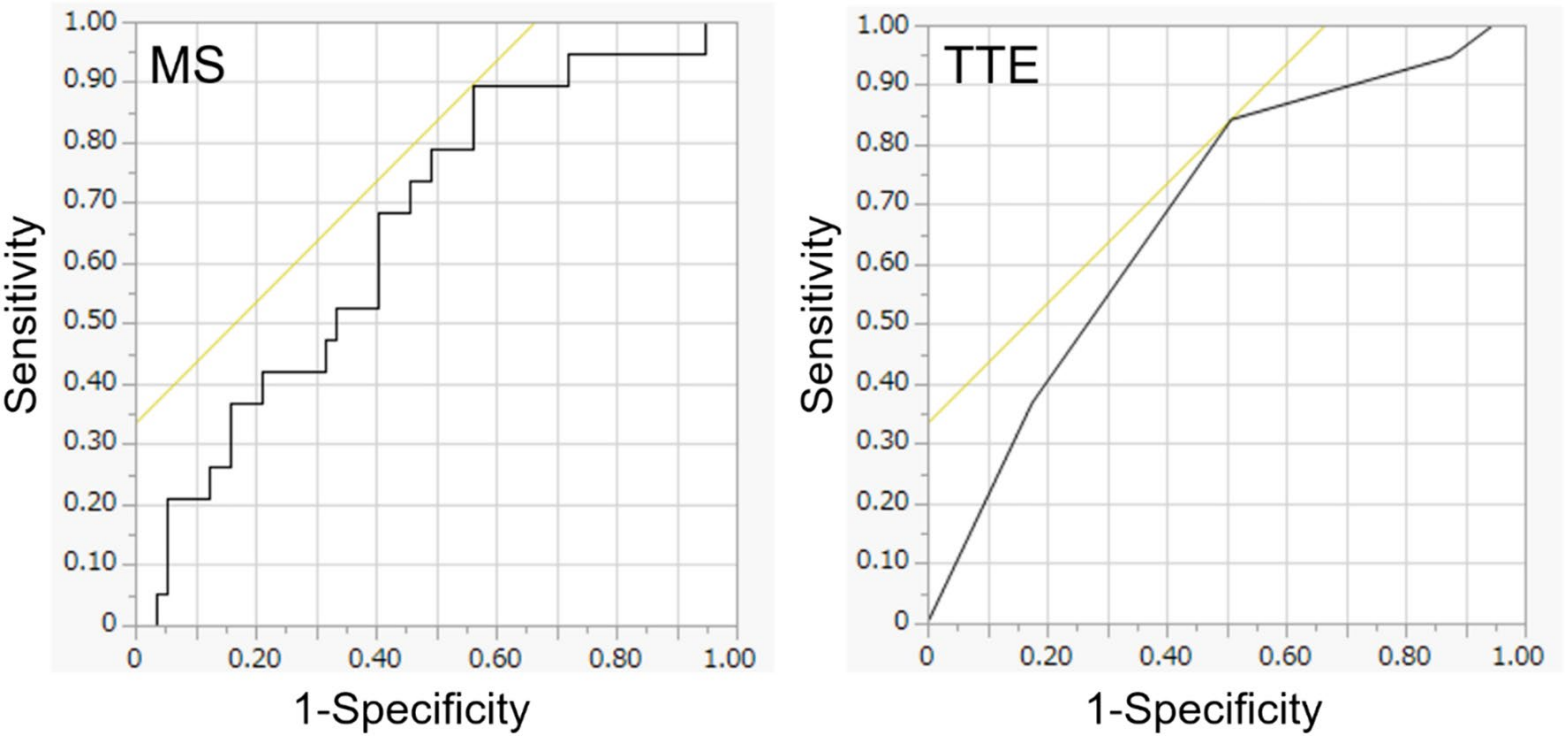
The receiver-operating characteristic (ROC) curve of MS and TTE values. In the ROC analysis, the area under the curve (AUC) for differentiating the low- and high-TILs groups was 0.661 for the MS and 0.685 for the TTE

The median MS values of the low- and high-TILs groups were 23.68 and 32.4, respectively. The MS of the high-TILs group was significantly higher than that of the low-TILs group (*p* = 0.037) (Table 3, Fig. 2). In the ROC analysis, the area under the curve (AUC) for differentiating the low- and high-TILs groups was 0.661. At a cut-off value of 22.63, the sensitivity and specificity for diagnosing high TILs were 89% and 44%, respectively (Fig. 3).

The median TTE values of the low- and high-TILs groups were both 5.8 (Table 3). However, the 25th, 75th, and 90th percentiles of the high-TILs group were shorter than those of the low-TILs group (2.9 vs. 5.8 at the 25th percentile, 5.8 vs. 8.7 at the 75th percentile, and 8.7 vs. 11.6 at the 90th percentile). The TTE values of the high-TILs group were significantly shorter than those of the low-TILs group in the Wilcoxon rank-sum test (*p* = 0.012) (Table 3, Fig. 2). In the ROC analysis, the AUC for differentiating the low- and high-TILs groups was 0.685. At a cut-off value of 5.8, the sensitivity and specificity for diagnosing high TILs were 82% and 49%, respectively (Fig. 3).

**Fig. 4:**
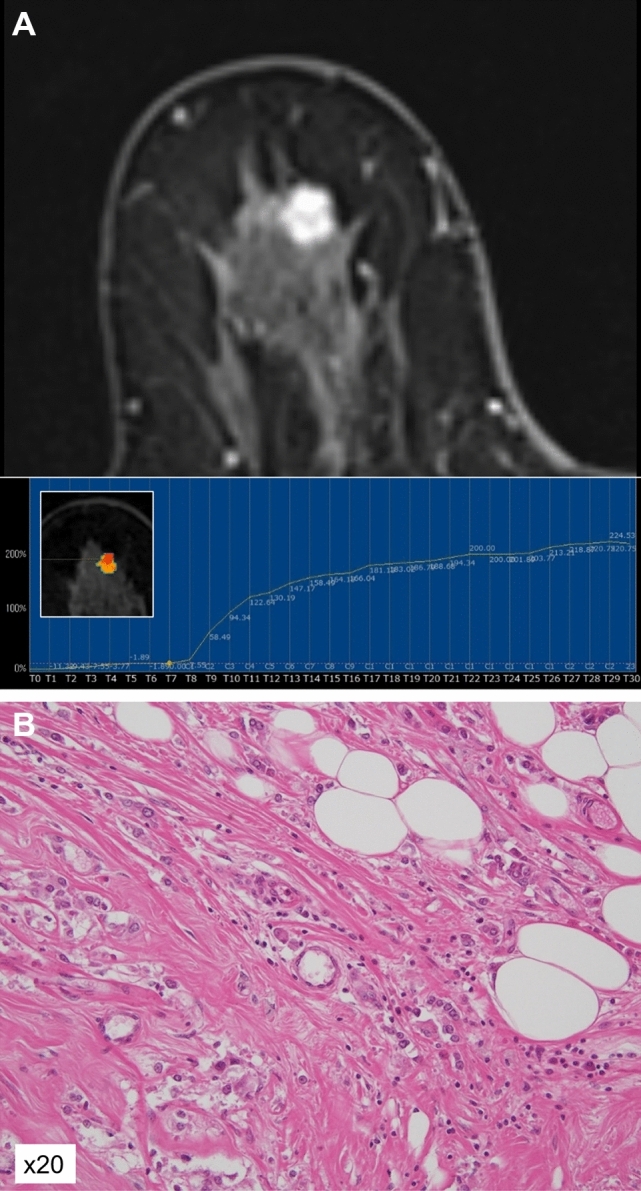
A 69-year-old woman with triple-negative breast cancer (TNBC) with low TILs. **A** The MS was 23.68%/sec and the TTE was 8.7 s. **B** Photomicrograph showing scattered TILs infiltration

**Fig. 5:**
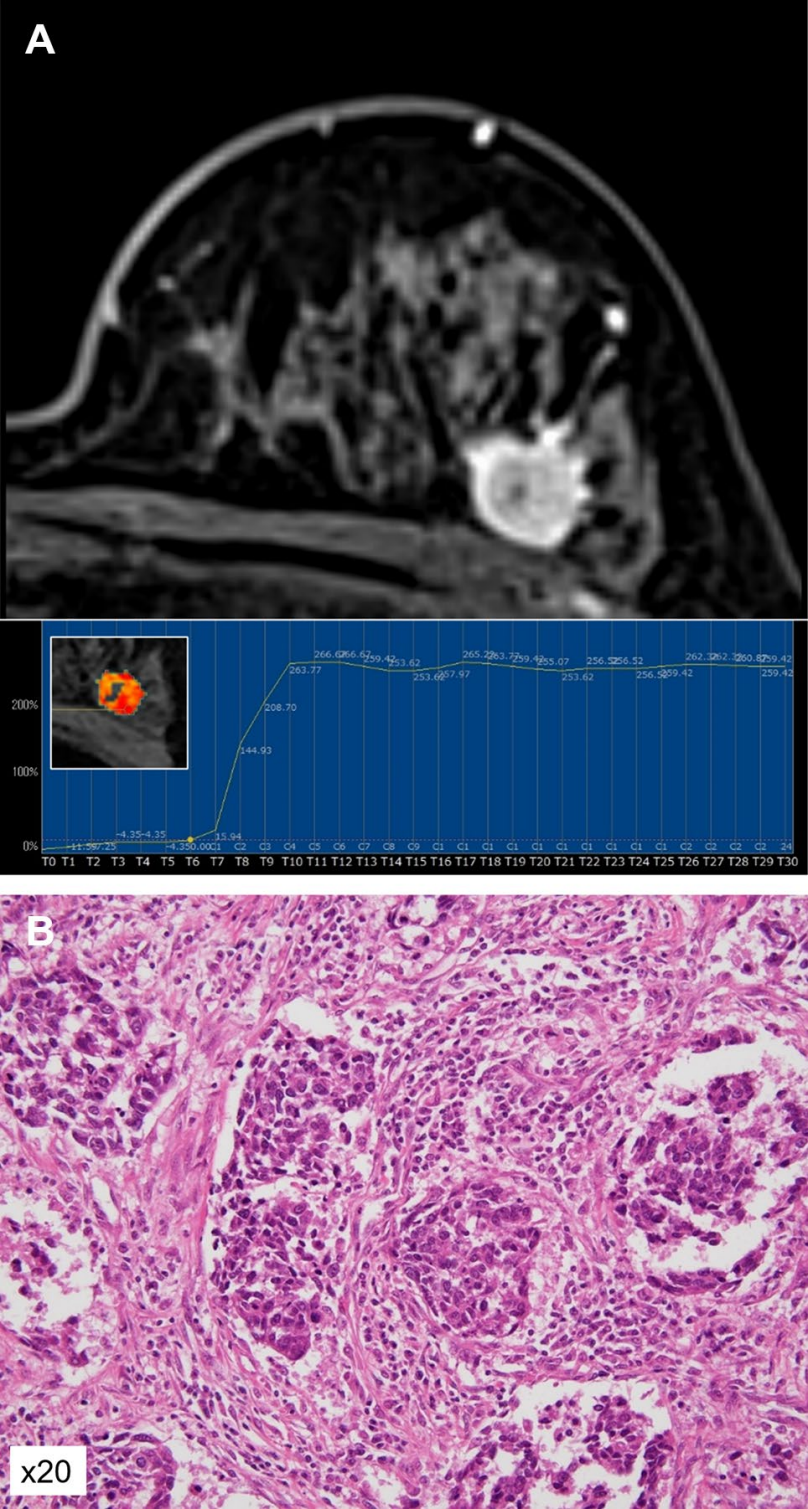
A 71-year-old woman with TNBC with high TILs. **A** The MS was 32.4%/sec and the TTE was 5.8 s. **B** Photomicrograph showing dense TILs infiltration

The relationships between the kinetic distribution of conventional DCE values and TILs status are presented in Table 4. The high-TILs group tended to have higher percentages of the washout pattern and lower percentages of the persistent pattern, but these differences were not significant. Representative cases from the low-TILs group and the high-TILs group are shown in Figs. 4 and 5.

**Table 4:** The relationships between the kinetic distribution of conventional DCE and TILs status

		Low TILs *n* = 57	High TILs *n* = 19	*P* value
Initial phase	Fast	97.91 (10.31–100)	99.71 (39.8–100)	0.0744
	Medium	2.09 (0–81.2)	0.29 (0–60.2)	0.0815
	Slow	0 (0–17.94)	0 (0–3.22)	0.3453
Delayed phase	Washout	11.34 (0–74.92)	22.28 (2.78–64.6)	0.082
	Plateau	64.82 (15.38–91.88)	66.15 (33.55–80.71)	0.8761
	Persistent	17.06 (0.98–83.76)	8.25 (0.91–29.15)	0.082

The data are percentages and are presented as median (range). DCE: dynamic contrast-enhanced

## Discussion

The results of our analyses demonstrated that the breast cancer with a high level of TILs had significantly higher MS and shorter TTE values compared to those with low level of TILs. In the ROC analyses, the AUCs for differentiating patients with low and high TILs were 0.661 for the MS and 0.685 for the TTE.

Several research groups have investigated TILs level using standard breast MRI, and according to some studies, breast cancer with high level of TILs tended to have higher ADC values, including all subtypes of breast cancer [9, 10]. In studies that examined only TNBC, breast cancer with high TILs tended to have a round shape, circumscribed margin, and lower percentage of the persistent pattern [7, 8]. In examinations of HER2-positive breast cancer, the high-TILs cases tended to have lower peak enhancement and peritumoral edema on T2WI [12, 13]. Several studies suggested that radiomics parameters are useful for predicting TILs status [14–16].

Thus, various MRI findings, including those obtained in the standard DCE studies, have been reported as predictors of TILs status. However, the targeted breast cancers vary from studies that included all subtypes (as in the present study) to those that are limited to TNBC and/or HER2-positive types. Of these, Ku et al. reported that in a series of TNBC, the high-TILs cases tended to have a lower percentage of the persistent pattern [8]. Although the results of our present analyses showed the same tendency, there were no significant between-group differences in the percentages of the kinetic parameters of the standard DCE sequence. In the study of Ku et al., only triple-negative cancer cases were included, cut-off value was 50%, and both 1.5 T and 3 T systems were included [8]. In our study, all subtypes were included, cut-off value was 10%, and only 3 T system was used. We think that these differences influenced the results. Further investigations with larger numbers of cases are necessary to resolve this.

The reported AUCs for differentiating low or high TILs status were 0.725–0.956 [8–10, 14–16], whereas the AUC values in the present study were 0.661 for the MS and 0.685 for the TTE, which were inferior to the previous studies. In this preliminary study, we investigated whether ultrafast DCE MRI alone is useful for predicting breast cancer TILs status, because (*i*) ultrafast DCE MRI is a relatively new method, and (*ii*) to the best of our knowledge, there are no published reports of direct investigations of ultrafast DCE MRI and TILs status. However, ultrafast DCE images can be acquired with short scan times. And this is a major advantage compared to standard breast MRI. If a radiomics study were to be conducted using ultrafast DCE MRI or a study is performed with combinations of ultrafast DCE MRI and other parameters, the diagnostic performance may increase. Here too, further investigations with larger numbers of cases are needed.

There are several study limitations to address. It was a single-institutional retrospective analysis and examined a relatively small number of lesions, particularly in HER2-positive and TN breast cancers. Good chemotherapy effects have been demonstrated in TILs-rich HER2-positive breast cancer and TNBC [3–5]. In recent years, there have been a report on the effects of TILs on ER-positive breast cancer [6]. However, this is not still clear in hormone receptor-positive breast cancer [5]. Each of these subtypes should also be investigated in ultrafast DCE imaging evaluations; we were not able to do this due to the small number of cases in this study. However, radiological imaging characteristics and breast cancer including all subtypes have been investigated [9–11, 14–16]. In addition, we used only the MS and TTE for evaluation, because only these two parameters are routinely calculated. Further research using other ultrafast DCE parameters such as the wash in slope (WIS) is needed.

In conclusion, the group of breast cancer with high levels of tumor-infiltrating lymphocytes (TILs) had higher maximum slope values and shorter time-to-enhancement values than those with low levels of TILs. Ultrafast DCE MRI would be useful for predicting breast cancer TILs status.
